# The impact of shade on whole-plant carbon allocation in a dominant East African tree sapling

**DOI:** 10.1093/aobpla/plaf039

**Published:** 2025-07-30

**Authors:** Gabriella M Mizell, Patrick D Milligan, Todd M Palmer, John Mosiany, John S Lemboi, Elizabeth G Pringle

**Affiliations:** Department of Biology, Program in Ecology, Evolution, and Conservation Biology, University of Nevada, Reno, 1664 N Virginia St., Reno, NV 89557, United States; Mpala Research Centre, Box 555, Nanyuki 10400, Kenya; Department of Biology, Program in Ecology, Evolution, and Conservation Biology, University of Nevada, Reno, 1664 N Virginia St., Reno, NV 89557, United States; Mpala Research Centre, Box 555, Nanyuki 10400, Kenya; Mpala Research Centre, Box 555, Nanyuki 10400, Kenya; Department of Biology, University of Florida, 876 Newell Dr., Gainesville, FL 32611, United States; Mpala Research Centre, Box 555, Nanyuki 10400, Kenya; Mpala Research Centre, Box 555, Nanyuki 10400, Kenya; Department of Biology, Program in Ecology, Evolution, and Conservation Biology, University of Nevada, Reno, 1664 N Virginia St., Reno, NV 89557, United States; Mpala Research Centre, Box 555, Nanyuki 10400, Kenya; Plants, Ecosystems & Climate

**Keywords:** tree-grass interaction, C allocation, non-structural carbohydrates, belowground reserves

## Abstract

Plasticity in resource allocation can be beneficial for plants under stress. In savannas, tree-grass competition forces tree saplings growing in the grass layer to compete for water, nutrients, and light. Savanna tree saplings are also vulnerable to fire and herbivory, which may favour investment in storage belowground to support regrowth aboveground. It is unclear whether carbon (C) limitation from grass shading similarly favours allocation belowground. Further, investigating how light reduction changes allocation by juvenile trees to above- and belowground biomass, storage, and defence can help us understand juvenile tree allocation strategies during ubiquitous C limitation. Using a screenhouse experiment, we evaluated the effects of shade on carbon allocation and leaf physiology in saplings of a dominant ant-acacia, *Acacia* (*Vachellia*) *drepanolobium*. We hypothesized that shade would induce greater belowground allocation by saplings to root growth and storage. Indeed, we found that shaded saplings had higher root mass fractions and higher concentrations of starch in their roots than plants in full sunlight. Plants in full sunlight, meanwhile, invested more in aboveground growth, with higher stem mass fractions than shaded plants. Shade did not affect leaf mass fraction, but plants in the shade had a lower leaf mass per area, higher stomatal conductance, and a higher maximum photosynthetic rate, indicating leaf-level adjustments that increased carbon capture under light limitation. These responses are consistent with possible adaptive allocation strategies that buffer the impacts of fire and herbivory, underscoring the essential role of belowground reserves for regrowth.

## Teaser

Tree-grass coexistence is a defining feature of savanna ecosystems, yet how young trees (saplings) persist within the grass layer remains unclear. Aboveground, saplings compete with grasses for light. Although shade can reduce photosynthesis and resulting carbon assimilation and associated energy available to a sapling, it is not known whether saplings adjust their energy allocation in response to grass shading. We measured above- and below-ground investment in growth and energy reserves (carbohydrates) in shaded and unshaded saplings of a common savanna tree and found that shaded saplings invested more in belowground growth and carbohydrates than saplings in full light. Higher belowground allocation by shaded saplings could enhance their long-term success if access to belowground resources and stored energy determine the potential for future aboveground growth.

## Introduction

Plant–plant interactions exert strong selection on plastic resource allocation strategies. For example, some plants that compete for soil nutrients belowground can allocate more resources to acquire the limiting nutrient at the expense of aboveground growth and seed mass (Gersani et al. 2001). Plasticity in resource-allocation strategies can be especially beneficial for plants that experience regular or predictable disturbances in their lifetime (Leung et al. 2020). For example, seasonal droughts may cause plants to invest in deeper roots (Padilla and Pugnaire 2007), and high herbivory pressure may periodically require higher investment in aboveground growth to replace shoots and leaves (Palacio et al. 2020). In savanna ecosystems, trees typically face regular disturbances in the form of grass competition, fire, and herbivory, which should favour such resource-allocation plasticity.

Tree-grass competition is common in savannas: trees and grasses compete belowground for water and nutrients and aboveground for light. These competitive interactions play a key role in structuring savanna vegetation (Bond 2008). Competitive pressure by grasses on trees increases during wet seasons, when grass biomass is the highest (February et al. 2013). Although grasses can suppress the growth of mature trees (Riginos 2009), grasses exert particularly strong impacts on juvenile trees. For example, in the Serengeti, grass competition can have a larger effect on seedling establishment than herbivory or drought (Morrison et al. 2019). Further, even once juvenile trees are established, they can be trapped in the grass layer, where higher fire, herbivory, and plant competition can suppress their recruitment to adulthood (Bond 2008, Staver et al. 2009). For example, in a Kenyan savanna, removing neighbouring grasses doubled sapling growth aboveground relative to controls (Riginos and Young 2007). Importantly, aboveground growth is only one aspect of the success of juvenile trees. Belowground growth, storage, and defence are other carbon sinks that are likely critical to sapling recruitment.

Carbon (C) allocation plays a central role in how plants plastically manage stress, like competition. Plant stress can cause C limitation, resulting in allocation trade-offs among sinks. Whole-plant biomass allocation provides some insight into such trade-offs. For example, following defoliation, oak saplings allocated more new C to canopy biomass at the expense of fine root production (Palacio et al. 2020). Allocation to storage of nonstructural carbohydrates (NSC) and defence can also be plastically regulated, particularly during times of stress (Chapin et al. 1990, Sala et al. 2012, Wiley and Helliker 2012). Starch is the most abundant NSC, and variation in NSC concentrations in response to environmental stress is driven mainly by changes in starch (Körner 2003, Hartmann et al. 2013, Puri et al. 2015). For example, C limitation caused by defoliation drove oak saplings to prioritize starch storage over growth (Wiley et al. 2013). In addition, burned seedlings have been found to use root starch reserves to resprout and supplement growth even after recovering leaf area (Schutz et al. 2009). Like herbivory and fire, plant–plant competition could affect the allocation or use of starch for savanna trees, but few studies have explicitly examined this possibility.

Juvenile trees in savannas are often shaded by grasses (Schutz et al. 2009, Morrison et al. 2019), resulting in competition for light. One way that plants may respond to shade is predicted by the functional equilibrium hypothesis (Brouwer 1963), which states that, under light limitation, biomass should be allocated aboveground to increase light capture, the limiting resource, at the expense of belowground growth. However, in addition to shade, juvenile trees in savannas usually encounter other aboveground stressors like fire and herbivory. To withstand such stress, savanna saplings under light competition may profit by investing in storage and growth belowground, which could support future growth aboveground when conditions are more favourable. Thus, the functional equilibrium hypothesis may not correctly predict allocation when multiple aboveground stressors are present. Further, there is evidence that allocation to starch increases juvenile-trees survival more than allocation to growth in light-limited environments. For example, in temperate forest tree seedlings, dark treatment led to greater declines in growth than in starch concentration, and reillumination led to starch refilling before growth resumed (Weber et al. 2018). Examining how allocation to biomass and starch storage changes under light limitation can help us understand how juvenile savanna trees survive prolonged C limitation.

In addition to investment in storage, shaded saplings may make leaf-level adjustments that enhance the efficiency of their limited C uptake. Within species, shaded plants can make plastic adjustments to their leaves, which is typically thinner leaves with a lower leaf mass per area (LMA) (Kitajima 1994). Reduced LMA can maximize light absorption (Givnish 1988) and minimize C loss through respiration. Leaf-level adjustments and increases in allocation to storage are not mutually exclusive, however, and may represent a whole-plant response to shade.

Here, we investigated C allocation priorities in a savanna tree sapling experiencing different light environments. *Acacia* (*Vachellia*) *drepanolobium* is a mono-dominant tree throughout much of its range in East African savannas, where it acts as a foundation species that increases nutrient cycling (Fox-Dobbs et al. 2010) and provides habitat and forage for herbivores (Riginos et al. 2015). *Acacia drepanolobium* is known for its ability to resprout (Okello et al. 2008), such that storage may be a critical component of its lifetime allocation strategy. Juvenile *A. drepanolobium* compete with grasses for establishment and can get trapped in the grass layer. To evaluate the effects of aboveground competition on C allocation, we experimentally imposed shade on *A. drepanolobium* saplings, evaluated proportional investments in biomass and starch storage, and tracked the relative allocation of pulsed ^13^CO_2_. We hypothesized that shade would induce saplings to allocate more C belowground, which could increase field survival by buffering the impacts of herbivory and fire. Based on this, we predicted that: (i) shading would induce plant changes to leaf traits to increase carbon capture per unit leaf area; (ii) shaded plants would have lower relative C allocation to stem growth than sun plants; and (iii) shaded plants would have higher relative C allocation belowground to roots and starch reserves than sun plants.

## Materials and methods

### Experimental setup

To test how simulated grass shading affects C allocation and assimilation in *A. drepanolobium* saplings, we conducted an experiment in a screenhouse at Mpala Research Centre (MRC; 0° 15′ 35.44″ N, 36° 53′ 53.36″ E) in central Kenya. The screenhouse was constructed of 25-mm mesh wire to keep animals out, and it produced minimal shade. Saplings were grown from seeds collected from four maternal trees in a black-cotton savanna 5 km from MRC. To randomize maternal effects, we mixed seeds into a single pool before planting. We planted seeds in 5 l pots in a soil mixture designed to approximate the maternal tree environment (85% black cotton vertisol and 15% sandy loam). Plants were watered to saturation (∼500 ml) every other day.

After growing in ambient light conditions in the screenhouse (∼1300–1700 µmol m^−2^ s^−1^) for 6 months, 160 saplings were randomly assigned to 1 of 2 light treatments: sun or shade. Saplings in the sun treatment were distributed throughout the 15 × 9 m screenhouse. Saplings in the shade treatment were placed under one of two shade cloths (2.5 × 5 m) on opposite sides of the screenhouse, with half of the shade saplings under each one. Shade cloths hung ∼0.5 m above the tallest plant and blocked ∼50% of ambient light (∼700 µmol m^−2^ s^−1^). Field microsite measurements from a Tanzanian savanna showed that grasses block between 30% and 70% of ambient light, depending on the season (Rugemalila et al. 2020), so we selected a value in the middle of this range. The shade treatment represents aboveground grass competition during the wet season, when grass biomass is the highest and water is not limiting. Saplings were in their respective light treatments for 14–18 days before pulse-labelling and physiology measurements began and the total light treatment time was between 15 and 46 days. Forty saplings were used only for measurements of LMA. We conducted biomass measurements on all of the remaining 120 plants, of which 60 were used for pulse-labelling and 60 were used as corresponding controls. Of the 120 plants we measured for biomass, we collected leaf physiology measurements on 100 plants and analysed NSC in 20 plants (see below).

### Leaf physiology measurements

To determine whether shading changed leaf physiology, we measured the maximum photosynthetic rate, stomatal conductance, and transpiration of 50 saplings per light treatment (*N* = 100). Leaf gas exchange measurements were collected at Days 15, 16, 17, 18, 19, 20, 21, and 25 after implementing the light treatment. We measured gas exchange using a CIRAS-3 Portable Photosynthesis System (PP Systems, Amesbury, MA, USA). Measurements were taken between 08:00 and 14:00 on one fully expanded leaf per plant, located ∼10 cm from an apical branch tip. The temperature of the leaf was controlled through the cuvette on the CIRAS-3 console, with a target temperature of 25°C. Leaf temperature at the time of measurement ranged from 25.6°C to 28°C according to the infrared thermometer inside the leaf cuvette. Because we alternated treatment groups during the measurement periods, we assume that this temperature drift contributed equally to the variance in our estimates of the leaf gas exchange rates for each treatment group. *Acacia drepanolobium* saplings at this age tend to saturate the photosynthetic rate at 1200 µmol e^−^ m^−2^ s^−1^ (P. Milligan, unpublished data), and cuvette conditions were maintained at 1500 µmol e^−^ m^−2^ s^−1^ using an LED artificial light source. Cuvette relative humidity was kept at 40%–70% (vapour pressure deficit of 1.2–2.2); *A drepanolobium* stomatal conductance remains relatively stable within this range (P. Milligan, unpublished data). Leaves were allowed to stabilize for ≥3 min in the leaf cuvette, and in rare instances required 5–7 min to (i) reach a leaf temperature of ∼28°C and (ii) stabilize stomatal conductance. When the ambient temperature was higher at midday, we often waited ∼7 min before recording gas exchange rates.

The 40 saplings reserved for measuring LMA were in their light treatments for 38 days before measurements occurred. From each of the 40 saplings, 5 fully expanded leaves were collected from branches that ended in nonlignified apical tips, from branch segments 5–10 cm from the tip. Leaves were placed onto a clean white sheet of paper printed with four 1-cm black bars to provide a visual reference for scale, flattened with a clean glass pane, and photographed with a 12.3 megapixel DSLR camera (Nikon D300) from directly above on a flat tabletop. All five leaves were collected into a single coin envelope and dried in a drying oven at 60°C for 48 h, and then weighed for total dry biomass. Live leaf area was traced and quantified in the program ImageJ (Schindelin et al. 2015) on a touchscreen Dell laptop with a PN350M stylus. LMA for each sapling was calculated as the total biomass of all five leaves divided by the total area of all five leaves.

### Pulse labelling

To track differential carbon allocation between light treatments in real time, we pulse-labelled saplings with ^13^CO_2_ using methods following Mizell et al. (2023), with the following modifications: we used a smaller chamber size (2.625 m^3^) and multiple plants (*n* = 20) per chamber. Plants from both shade and sun treatments experienced full sun during the pulse-labelling, which lasted 3 h, to allow for sufficient and timely uptake of ^13^CO_2_. Briefly, plants were enclosed in the chamber, which was made of clear greenhouse plastic hung over interlocking metal poles and closed with spring clamps. Four battery-operated fans were placed in the top corners of the chamber to circulate the ^13^CO_2_. ^13^CO_2_ was pumped into the chamber by reacting labelled sodium bicarbonate (NaH^13^CO_3_) with acetic acid and CO_2_ concentration was continuously monitored using an infrared gas analyser (IRGA). Temperature, humidity, and photosynthetically active radiation were also monitored throughout the labelling period to compare them with ambient conditions and to ensure that deviations from ambient conditions were consistent among labelling groups (Supplementary Table S1).

In total, we labelled 30 saplings per light treatment (*N* = 60) in 3 groups, with 10 sun plants and 10 shade plants in the chamber in each labelling event over 3 consecutive days in June 2022. Plants from each light treatment were randomly chosen for each labelling event. Each labelling event lasted for 3.2 h on average, ending when all of the ^13^CO_2_ was added and CO_2_ in the labelling tent declined to an IRGA reading of ∼200 ppm (which corresponded to an actual CO_2_ concentration ∼385 ppm, due to the inefficiency of the IRGA at reading ^13^CO_2_). Following labelling, plants were placed back into their respective light treatments. There were 60 unlabelled plants (half in each light treatment) that served as controls to assess the success of pulse-labelling. For the chase period, saplings were randomly assigned to destructive sampling at one of the following six time points after labelling: 1, 2, 4, 7, 14, or 28 days, such that five control and five labelled plants from each light treatment were sampled at each time point (20 plants × 6 time points = 120).

### Tissue collection and isotope analysis

To determine the relative allocation of labelled C between light treatments, we collected leaves, stem tissue, root tissue, and root nodules from each plant prior to harvesting total biomass. Tissues were collected as follows: four to five fully expanded and undamaged leaves from various parts of the plant were clipped, a 3-cm lignified stem tip was clipped from a haphazardly chosen branch, a 3-cm section of coarse root (>2 mm diameter) was clipped from a lateral root immediately adjacent to the tap root, and four to five root nodules were chosen haphazardly from the root system. Sampled tissues were placed immediately on ice, microwaved within 3 h of collection to stop enzymatic activity (Landhäusser et al. 2018), stored at −20°C, and then dried at 65°C for 48 h. Dried tissues were stored in silica until they were homogenized with 2.8-mm stainless-steel grinding balls in a tissue lyser. Homogenized samples (1–2 mg) were analysed for *δ*^13^C at the UC Davis Stable Isotope Facility, using a PDZ Europa ANCA-GSL elemental analyser interfaced with a PDZ Europa 20-20 isotope ratio mass spectrometer.

To determine the total amount of ^13^C in each sampled sink, we scaled the *δ*^13^C from each 1–2 mg sample to the amount of ^13^C in each sink (0.0001–0.31 g), using our biomass measurements (see below). All scaled C sinks were added together to estimate total ^13^C in each plant. The ^13^C in each sink was divided by the total ^13^C in the plant to obtain the isotope mass fraction.

### Biomass measurements

To determine differences in biomass allocation between light treatments, each sapling was harvested entirely when its samples were collected for ^13^C analysis. Saplings were removed from their planting bags, and we removed soil from the root systems by carefully washing them in soapy water with baking soda. Roots, leaves, and stems, were separated and dried in a solar oven for ∼14 days. We weighed dry roots, stems, and leaves separately to determine their individual biomasses, and we summed these masses together to obtain total plant biomass. The biomass of each tissue was divided by the total biomass of the plant to obtain root, leaf, and stem mass fractions. Root nodules were not included as a separate biomass sink because after drying with roots, nodules shrank and were difficult to distinguish from the roots.

### Nonstructural carbohydrate analysis

We measured starch concentrations in the saplings that were harvested 28 days after pulse labelling (*N* = 20). Using a subsample of the tissues collected for the stable isotope analysis, we measured starch in stems and roots using enzyme quantification (Landhäusser et al. 2018). In brief, sugars were removed from ground plant material with 80% ethanol at 90°C. In the remaining pellet, starch was converted to glucose by adding α-amylase followed by amyloglucosidase. Then, it was converted to gluconate using hexokinase (glucose assay reagent, Sigma Aldrich). The amount of glucose (converted from starch) was determined photometrically at 340 nm. A standard curve was used to quantify the glucose concentration in each sample.

### Statistical analysis

All analyses were run in R (version 4.1.1). To assess the effects of shading on sapling leaf physiology, biomass allocation, new C allocation, and starch concentrations, we used linear models in the glmmTMB package (Brooks et al. 2017), unless otherwise noted. Details for each model are described below. We report means and standard errors of the response variables.

To test whether the shading treatment reduced total carbon assimilation, we compared total sapling biomass between the two light treatments (*N* = 120) and included the number of days since treatments began as a fixed effect. To test whether the saplings had successfully assimilated the ^13^C label, we modelled *δ*^13^C of all plant material as the response in labelled and control saplings (*N* = 120), using labelling treatment, number of days since labelling, and their interaction as fixed effects. The interaction was included to account for our expectation that the *δ*^13^C in labelled plant tissue would decrease over time, whereas the *δ*^13^C in control plants would remain constant.

To evaluate how the light treatments affected leaf physiology, we modelled light treatment and the number of days in the light treatment before measurements were taken as fixed effects for each of the following leaf-level responses: maximum photosynthetic rate (*A*_max_), stomatal conductance (*g*_s_), and transpiration (*E*). Because LMA measurements were conducted on a single day, we used a linear model to assess its response to the fixed effect of light treatment.

Measurements of relative biomass (mass fraction) show differences in structural C investment between the light treatments, whereas relative ^13^C mass fractions (‘isotope mass fractions’, or *δ*^13^C scaled to biomass) show differential allocation of recent photoassimilates to each sink. We examined these measurements together to determine whether C allocation matched apparent investment in biomass. To determine whether light treatment affected biomass and isotope mass fractions in each tissue, we modelled light treatment and number of days since light treatment began as fixed effects for each of the following responses: leaf mass fraction, stem mass fraction, root mass fraction, leaf isotope mass fraction, stem isotope mass fraction, and root isotope mass fraction. We also modelled *δ*^13^C using a linear model with light treatment and days since the light treatment began as fixed effects. We did not include time since labelling as a fixed effect in this model because the fixed effect of time spent in the light treatment integrates both days since labelling and variation among the three labelling groups in days spent in light treatments. To examine whether changes in above- and below-ground biomass were due to relative differences among different-sized tissues rather than to treatment effects, we used the analysis of covariance: the proportion of the total biomass that was above or belowground, the light treatment, and their interaction were used as factors to predict total biomass (Phillips and Leger 2015).

To determine how light treatment affected starch storage, we modelled the following responses separately: concentration of starch in stems [% sugar (weight of sugar/dry weight of plant sample)], concentrations of starch in roots (%), total starch (g) in roots, and total plant starch (g). Each model was a linear mixed effect model, with light treatment as the fixed effect and plant ID (unique identified number) as a random intercept.

## Results

### Treatment success

**Figure 1.**
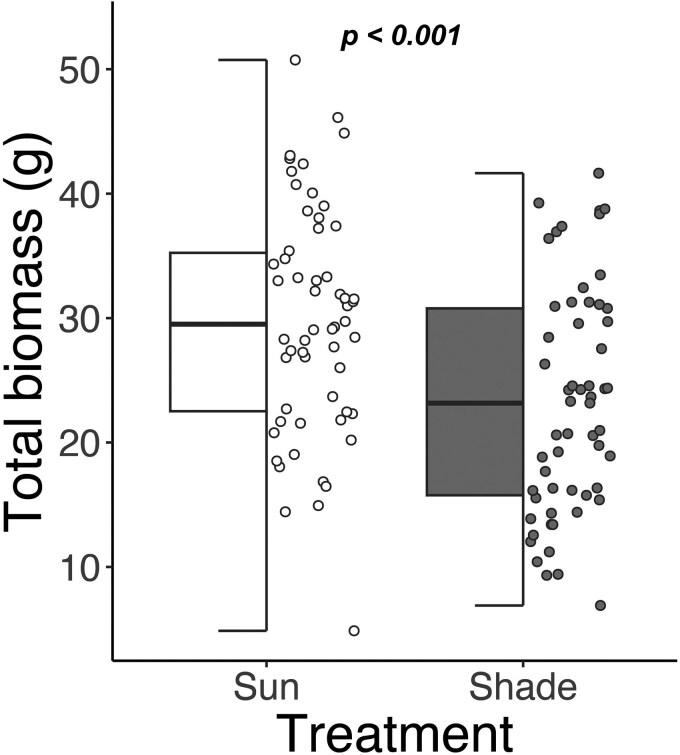
Total biomass (g) of *A. drepanolobium* saplings in sun and shade treatments, pooled across sampling time points (*n* = 20 samples at six timepoints). The partial boxplots show medians, interquartile range, and outliers. The *P*-values are based on a linear model with total biomass as the response and light treatment and days in light treatment as fixed effects.

Sun plants had a higher total biomass than shade plants (30.68 ± 1.24 versus 23.71 ± 1.23 g, *Z* = 4.00, *P* < .001, Fig. 1), indicating that the light treatment was sufficient to impact C assimilation. Total plant biomass did not change over the sampling period (*Z* = 0.47, *P* = .64). Plants labelled with ^13^CO_2_ had significantly higher amounts of *δ*^13^C in their tissues (Supplementary Fig. S1), indicating successful uptake of the label. Variation in *δ*^13^C among the unlabelled plants in the two light treatments was small (∼1‰ versus ∼65‰ in labelled plants), so the remaining ^13^C statistical analyses include only the labelled plants.

### Leaf physiology

Stomatal conductance and transpiration were higher in shade plants than in sun plants (420.93 ± 26.83 versus 333.15 ± 24.59 mmol m^−2^ s^−1^, *P* = .015 and 7.38 ± 0.40 versus 5.99 ± 0.38 mmol H_2_O m^−2^ s^−1^, *P* = .01, respectively). Similarly, shade plants had a 12% higher maximum photosynthetic rate than sun plants (15.74 ± 0.56 versus 14.07 ± 0.67 µmol CO_2_ m^−2^ s^−1^, *Z* = −1.9, *P* = .056). LMA was lower for shade plants than for sun plants (8.94 ± 0.29 versus 11.19 ± 0.65 g cm^−2^, *Z* = 3.76, *P* < .001); i.e. shade plants had thinner leaves. None of the leaf physiology responses varied with days in light treatment.

### Biomass and ^13^C investment

**Figure 2.**
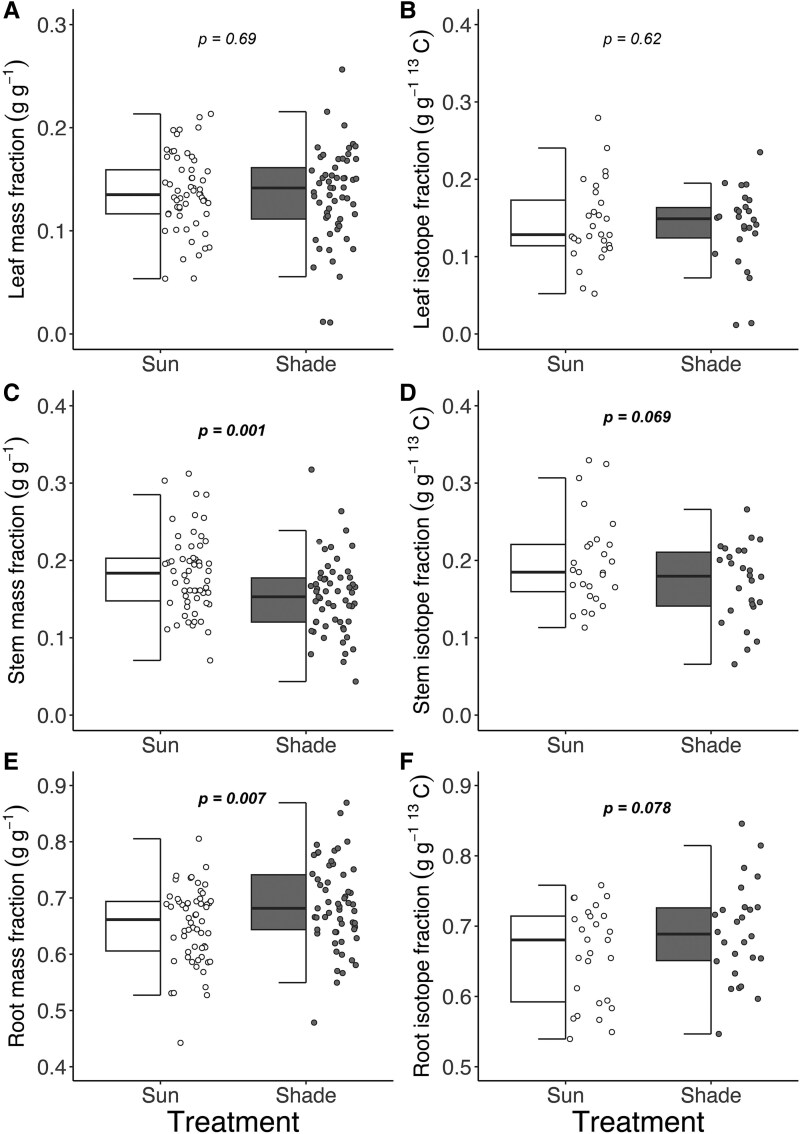
Biomass and isotope mass fractions in leaves (a and b), stems (c and d), and roots (e and f) in saplings that experienced sun or shade treatment. (a, c, and e) The fraction of the total biomass that is in leaves, stem, and roots, respectively. (b, d, and f) The fraction of total ^13^C found in leaves, stems, and roots, respectively. The partial boxplots show medians, interquartile range, and outliers. The *P*-values are based on a linear model with mass or isotope mass fraction as the response and light treatment and days in light treatment as fixed effects.

There was no difference in leaf mass fraction or leaf isotope mass fraction between plants in the two light treatments (Fig. 2a and b) and neither declined over time. Shade plants showed a 16% lower stem mass fraction than sun plants (Fig. 2c; 0.183 ± 0.007 versus 0.153 ± 0.006, *Z* = 3.21, *P* < .01) and a 13% lower stem isotope mass fraction (Fig. 2d; 0.197 ± 0.011 versus 0.171 ± 0.010, *Z* = 1.82, *P* = .069), with no changes over time. Conversely, shade plants had a 6% higher root mass fraction than sun plants (Fig. 2e; 0.651 ± 0.009 versus 0.687 ± 0.01, *Z* = −2.71, *P* < .01) and a marginally higher root isotope mass fraction (Fig. 2f; 0.66 ± 0.013 versus 0.691 ± 0.014, *Z* = −1.761, *P* = .078), with no changes over time.

Unlike the patterns in isotope mass fractions (those scaled to biomass), shaded plants had a higher *δ*^13^C in leaves (Supplementary Fig. S1a; Z = −2.73, *P* < .01) and *δ*^13^C declined over sampling time (Supplementary Fig. S1a; *Z* = −3.83, *P* < .01). In stems, there was a trend towards higher *δ*^13^C in sun plant stems than shaded plants (Supplementary Fig. S1b; *Z* = −1.59, *P* = .011) and *δ*^13^C declined over time (Supplementary Fig. S1b; *Z* = −2.43, *P* = .015). There was no difference in root *δ*^13^C between treatments or over time (Supplementary Fig. S1c). There was no relationship between total biomass and the proportions of biomass above- or below-ground (*F* = 0.02, *P* = .88) or the interaction between light treatment and proportions of biomass above and belowground (*F* = 0.82, *P* = .37), indicating that the higher apparent investment by shade plants in belowground biomass was due to the effects of the light treatment rather than to their lower overall biomass.

### Starch storage

**Figure 3.**
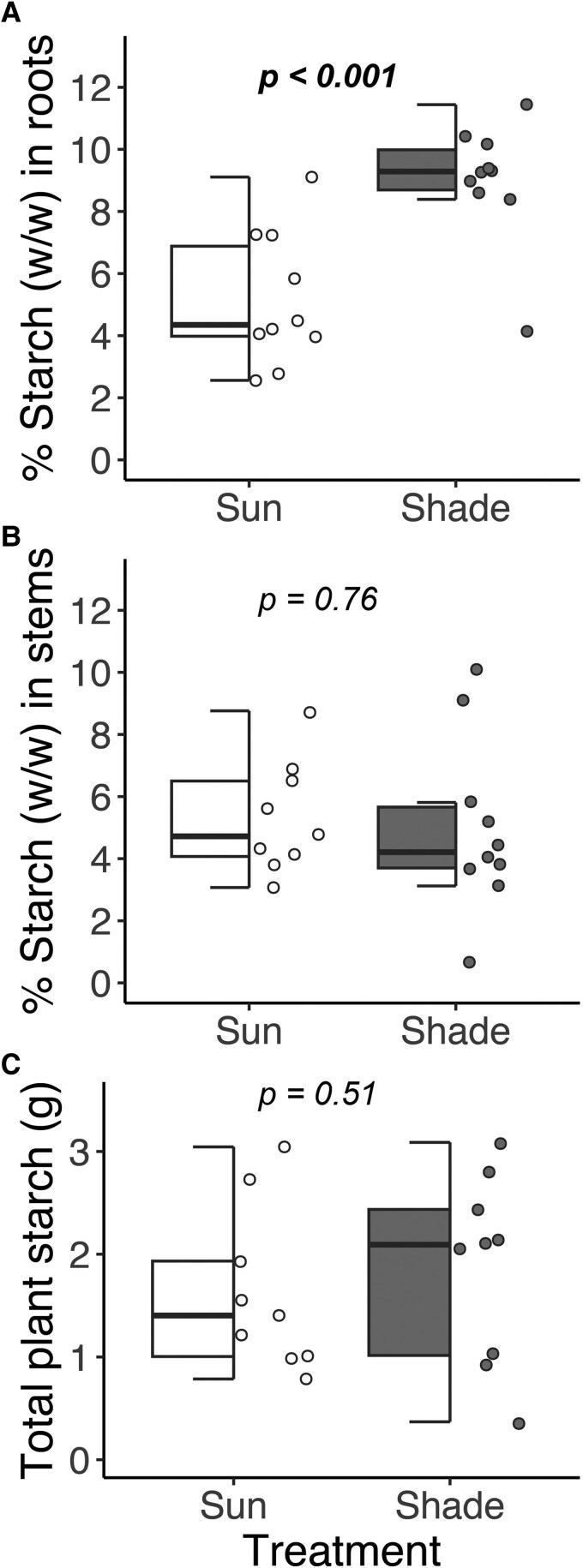
Starch in saplings that experienced sun or shade. (a) Concentration of starch in roots (mg/mg), (b) concentration of starch in stems (mg/mg), and (c) the total amount of starch (g) in whole plants. The partial boxplots show median, interquartile range, and outliers. The *P*-values are based on a linear model with concentration or total amount of starch as the response, light treatment as the predictor, and plant ID as a random effect.

Shade plants had a 75% higher starch concentration in their roots than sun plants (9.01 ± 0.61% versus 5.15 ± 0.68%, *Z* = −4.46, *P* < .001, Fig. 3a). There was no difference in the starch concentration in stems between shade and sun plants (Fig. 3b). After scaling starch concentrations to total biomass, there was a trend towards higher total starch in shade plants (Fig. 3c), but this difference was not statistically significant (1.88 ± 0.30 vs. 1.63 ± 0.26 g, *Z* = −0.66, *P* = .51).

## Discussion

In this study, we investigated how shade affects allocation to biomass and C storage in saplings of a dominant savanna tree. Overall, we found that *A. drepanolobium* saplings shift investment from stem growth to root growth and NSC storage while simultaneously adjusting leaf traits, which may optimize light capture under shaded conditions. *Acacia drepanolobium* saplings thus behave in a manner contrary to the functional equilibrium hypothesis: rather than investing in aboveground structures to capture more of the limiting light resource, *A. drepanolobium* saplings invest in structures that may contribute to the ability to withstand shade and survive in the longer term. Storage investments belowground could be particularly favoured by strong aboveground stressors like fire and herbivory in savanna ecosystems.

In our study, the active allocation of ^13^C-labelled photosynthates followed the same pattern as biomass allocation, but only when we scaled the *δ*^13^C to the biomass of each sink. Per-unit differences at the scale of *δ*^13^C do not necessarily indicate the direction of differential allocation among sinks if the treatment causes differential allocation to biomass, as our light treatment did here. For example, although the isotope mass fractions showed a relatively low investment in stems by shade plants in favour of roots, the *δ*^13^C signatures, by contrast, were higher in the stems of shade plants. Similarly, scaling NSC concentrations to the biomass of whole sinks has been shown to be important when evaluating whole-tree carbon status (Hesse et al. 2021). Two caveats to scaling labelled carbon are: (i) the linear scaling of *δ*^13^C to the whole sink may be misleading if there is unequal distribution of the label throughout the tissue (Epron et al. 2012); (ii) it may be challenging to scale isotopic fractions accurately in adult trees if it requires an accurate estimation of biomass. Nevertheless, surprisingly few labelling studies that do measure the biomass of each sink have also tried scaling *δ*^13^C values to these biomasses.

The shade response of young savanna woody plants appears to be species specific (Hoffmann 1996, Issifu et al. 2019). For example, *Acacia (Vachellia) tortilis* has lower establishment rates in the shade than in open areas (Smith and Shackleton 1988), whereas *Acacia (Senegalia) mellifera* is able to germinate and survive in both open and shaded areas equally (Kraaij and Ward 2006). Although we did not measure the establishment or survival of *A. drepanolobium* in the field in different light environments, we found that C allocation patterns in *A. drepanolobium* saplings were consistent with shade tolerance. Moreover, the pattern of C allocation we observed in this experiment may represent an adaptive strategy given the other stressors facing juvenile trees in the savanna, particularly given that *A. drepanolobium* is a strong resprouter following herbivory or fire disturbance (Okello et al. 2008).

Although our results are consistent with our hypothesis that belowground allocation under low light should offer a selective advantage under conditions of regular aboveground stress from fire and herbivory, our results contrast with the functional equilibrium hypothesis (Brouwer 1963). Evidence for this hypothesis in woody savanna species, which frequently recruit in the grass understory, is mixed. Consistent with our findings, Issifu et al. (2021) found that savanna seedlings had a slightly higher root mass fraction when shaded by grasses in a field experiment. Similarly, relative leaf investment was not different in that study between shade and sun seedlings. However, specific stem length (not biomass) was higher in the shade in the Issifu et al. (2021) study, which can indicate shade avoidance (Schmitt et al. 1999). Our results contrast more generally with another greenhouse study examining the impact of shade on seedlings from other species in the genus *Acacia* (*Vachellia*) in Tanzania. In that study, *A. tortilis* and *Acacia robusta* seedlings in shade (25% full sunlight) developed lower root mass fraction and higher leaf and stem mass fractions than seedlings in full sunlight (Rugemalila et al. 2020), the opposite of what we found in *A. drepanolobium*. One potentially important difference between that study and ours is the savanna soils to which each acacia species are adapted. *Acacia drepanolobium* grows in ‘black-cotton’ savannas, where the soil is a clay-rich (≥50%) vertisol, whereas the seeds of *A. tortilis* and *A. robusta* came from multiple sites with an average clay content of 24% (T. M. Anderson, personal communication). The high clay content of black-cotton soils causes shrink-swell dynamics during seasonal rains and dry periods, creating a physically stressful environment for plant roots (Whitmore and Whalley 2009). Because root diameter positively correlates with root biomass and tensile resistance (Blouin et al. 2007, Yang et al. 2016, Milligan et al. 2023), plants faced with C limitation in these stressful soils may need to ensure that their roots are thick enough to avoid breakage (Pringle et al. 2016). In addition, our finding that shade favours higher concentrations of starch in the roots may also favour bigger roots, which have additional storage function in some species (Issifu et al. 2019, Boonman et al. 2020).

Ontogenetic differences in response to shade could also be important and help to explain contrasting results among studies. Plant traits can shift rapidly during early development, shifting from resource-acquisitive to more conservative in mere months (Havrilla et al. 2021). In particular, younger plants may be more susceptible to mortality from C limitation than older plants, making it more important to have a light acquisitive strategy. Three-year-old Scots pine seedlings had a higher survival rate than 2-year-olds following simulated herbivory, shade, and both combined, presumably because older plants had more reserves (Hódar et al. 2008). Such ontogenetic changes could also help explain the differences between the responses of *A. drepanolobium* in this study and those of the other East African acacias in Rugemalila et al. (2020). The *A. tortilis* and *A. robusta* seedlings in that study were placed in their respective light treatments immediately after germination and remained there for 3 months. In contrast, our plants were 7–8 months old, at which point sufficient carbon gain may have occurred in the plant's lifetime to employ a more conservative, stress-tolerating strategy. Another contrast in timing may explain why we found higher starch storage in the roots of shaded plants, whereas Issifu et al. (2021) found no differences in the concentration of root NSCs proportional to biomass and lower total NSCs in shaded plants. In that study, seedlings in the shaded plots received 11% relative light for 7 months, whereas seedlings in our experiment received ∼50% relative light for 3–6 weeks. It is possible that more severe C limitation drains NSC reserves.

A few caveats bear mention. Our study investigated the impact of aboveground grass competition on its own, but the influence of grasses on saplings may change in other contexts. For example, grass shading can be facilitative by concealing saplings from herbivores (Riginos and Young 2007) or by preventing evaporative water loss in dry soils (Palmer et al. 2017). Additionally, it is possible that our light treatment resulted in unintended lower soil temperature in the shade than in the sun, which cannot be separated from the effects of shade.

Our study adds to the growing evidence that trees can actively allocate C to storage, which could offer a selective advantage for saplings trapped in the grass layer if they can later use that starch to grow rapidly beyond the grass layer (Bond 2008, Staver et al. 2009, Wakeling et al. 2011). Such responses are likely species specific and depend on the ability to resprout, as well as other stressors, such as herbivory and fire, in the system. Specifically, stored starch could potentially supplement current photosynthesis for rapid growth during periods of release from C limitation (Schutz et al. 2009) and allow faster transition into adulthood (Wigley et al. 2009). Long-term studies tracking whole-plant C allocation and temporal NSC dynamics in the field at various ontogenetic stages are needed to determine whether adaptive allocation strategies exist in savanna trees as well as the dependence of such strategies on variable ecological conditions.

## Supplementary Material

plaf039_Supplementary_Data

## Data Availability

The data underlying this article are available on Dryad Digital Repository: https://doi.org/10.5061/dryad.bvq83bknb.
