# Supplementary material for: The impact of shade on whole-plant carbon allocation in a dominant East African tree sapling
Source: AoB Plants. 2025 Jul 30;17(4):plaf039. doi: 10.1093/aobpla/plaf039 (PMC12380175; doi:10.1093/aobpla/plaf039)
Supplement: plaf039_Supplementary_Data [file plaf039_supplementary_data.pdf]

Electronic supplementary material

Title: The impact of shade on whole-plant carbon allocation in a dominant East African tree sapling

Gabriella M. Mizell[1,2*], Patrick D. Milligan[1,2], Todd M. Palmer[2,3], John Mosiany[2], John S. Lemboi[2], Elizabeth G. Pringle[1,2]

[1]Department of Biology, Program in Ecology, Evolution, and Conservation Biology, University of Nevada, Reno, NV, USA
[2]Mpala Research Centre, Box 555-10400, Nanyuki, Kenya
[3]Department of Biology, University of Florida, Gainesville, FL, USA

*****correspondence: gmizell@unr.edu

Table S1: The average and maximum temperature and relative humiditiy (RH) in the labeling tent and ambient conditions during ^{13}C labeling.

			In labeling tent				Ambient			
Labeling group	Labeling date	Time in tent (hours)	Mean temp (°C)	Max temp (°C)	Mean RH (%)	Max RH (%)	Mean temp (°C)	Max temp (°C)	Mean RH (%)	Max RH (%)
1	30 June 2022	3.1	23.0	29.8	74.9	81.2	20.4	24.3	50.0	74.4
2	1 July 2022	3.3	25.3	34.9	71.3	81.7	20.9	24.8	49.2	77.1
3	4 July 2022	3.07	23.9	32.9	76.5	90.1	21.3	24.7	44.9	67.8

Figure S1

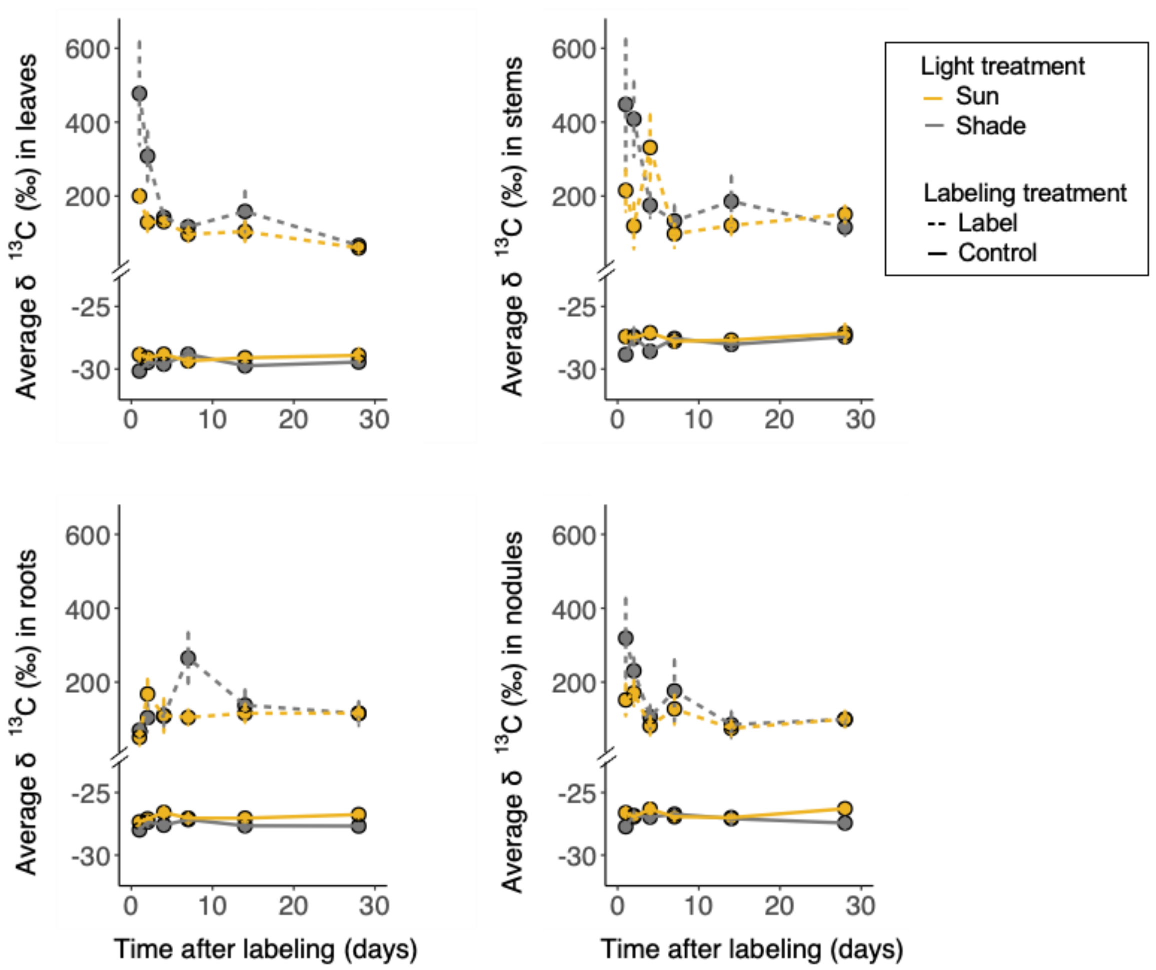

Figure S1: The average of $\delta\ ^{13}C$ (‰) in a) leaves, b) stems, c) roots and d) nodules. Colors represent light treatment and line types indicate labeling treatment. Means and standard errors are shown for plants sampled at each time point after labeling. Note the y-axis break.
